# Postpartum weight retention and the early evolution of cardiovascular risk over the first 5 years after pregnancy

**DOI:** 10.1186/s12933-024-02184-4

**Published:** 2024-03-18

**Authors:** Caroline K. Kramer, Chang Ye, Anthony J Hanley, Philip W Connelly, Mathew Sermer, Bernard Zinman, Ravi Retnakaran

**Affiliations:** 1 Leadership Sinai Centre for Diabetes, University of Toronto, Mount Sinai Hospital, 60 Murray Street, Suite L5-025, Mailbox-21, Toronto, ON M5T 3L9 Canada; 2 Division of Endocrinology, University of Toronto, Toronto, Canada; 3 Lunenfeld-Tanenbaum Research Institute, Mount Sinai Hospital, Toronto, Canada; 4 Department of Nutritional Sciences, University of Toronto, Toronto, Canada; 5 Keenan Research Centre for Biomedical Science of St. Michael’s Hospital, Toronto, Canada; 6 Department of Laboratory Medicine and Pathobiology, University of Toronto, Toronto, Canada; 7 Department of Obstetrics and Gynecology, Mount Sinai Hospital, Toronto, Canada

**Keywords:** Postpartum weight retention, Pregnancy, Cardiovascular risk, Lipids, Diabetes

## Abstract

**Background:**

The cumulative effect of postpartum weight retention from each pregnancy in a woman’s life may contribute to her risk of ultimately developing type 2 diabetes and cardiovascular disease. However, there is limited direct evidence supporting this hypothesis. Thus, we sought to characterize the impact of postpartum weight retention on the trajectories of cardiovascular risk factors over the first 5-years after pregnancy.

**Methods:**

In this prospective observational cohort study, 330 women (mean age 35.7 ± 4.3 years, mean pre-pregnancy body mass index 25.2 ± 4.8 kg/m^2^, 50.9% primiparous) underwent serial cardiometabolic characterization (anthropometry, blood pressure, lipids, oral glucose tolerance test, insulin sensitivity/resistance (Matsuda index, HOMA-IR), C-reactive protein (CRP), adiponectin) at 1-year, 3-years, and 5-years postpartum. Based on the magnitude of weight change between pre-pregnancy and 5-years postpartum, they were stratified into the following 3 groups: weight loss (*n* = 100), weight gain 0–6% (*n* = 110), and weight gain ≥ 6% (*n* = 120).

**Results:**

At 1-year postpartum, cardiovascular risk factors did not differ between the groups. However, an adverse risk factor profile progressively emerged in the weight retention groups at 3- and 5-years. Indeed, after covariate adjustment, there was stepwise worsening (from the weight loss group to weight gain 0–6% to weight gain ≥ 6% group) of the following cardiovascular risk factors at 5-years: triglycerides (*p* = 0.001), HDL (*p* = 0.02), LDL (*p* = 0.01), apolipoprotein-B (*p* = 0.003), Matsuda index (*p* < 0.0001), HOMA-IR (*p* < 0.0001), fasting glucose (*p* = 0.07), and CRP (*p* = 0.01). Moreover, on logistic regression analyses, weight gain ≥ 6% emerged as an independent predictor of pre-diabetes/diabetes at 5-years (adjusted OR = 3.40, 95%CI: 1.63–7.09).

**Conclusion:**

Postpartum weight retention predicts trajectories of worsening cardiovascular risk factors and glucose intolerance over the first 5-years after delivery, consistent with its postulated contribution to future vascular disease in women.

## Introduction

There is currently considerable interest in the intersection of pregnancy, body weight and future risk of cardiovascular disease (CVD) [1–3]. Indeed, maternal weight gain over the 9-months of gestation is an anticipated feature of normal pregnancy. However, in the years after delivery, ongoing retention of the weight accrued during pregnancy can have adverse consequences for a woman’s future weight trajectory [4–10]. Notably, a woman’s weight at 1-year postpartum is a stronger predictor of her likelihood of being overweight 15 years later than the weight that she gained during the pregnancy [9]. The significance of these observations is further underscored by the recognition that overweight/obesity is associated with increased risks of developing type 2 diabetes (T2DM) and CVD [11]. Accordingly, it has been postulated that the cumulative effect of postpartum weight retention from each pregnancy in a woman’s life may contribute to her risk of ultimately developing these conditions [10, 12, 13]. However, there is limited direct evidence supporting this hypothesis. Thus, in this context, our objective was to characterize the relationship between postpartum weight retention and the trajectories of cardiovascular risk factors over the first 5-years after pregnancy.

## Methods

This study was performed in the setting of a prospective observational cohort wherein pregnant women have been recruited at our institution to undergo serial cardiometabolic characterization in the years after delivery. The study protocol has been previously described in detail [14]. In brief, all pregnant women at our centre are screened for gestational diabetes mellitus (GDM) by 50 g glucose challenge test (GCT) at 24–28 weeks gestation, followed by oral glucose tolerance test (OGTT) in those in whom the GCT is abnormal (plasma glucose ≥ 7.8 mmol/l at 1-hour post-challenge). Study participants were recruited either before or after the GCT, with the latter recruitment serving to enrich the study population for those with dysglycemia in pregnancy. For this study, all participants completed a 3-hour 100 g OGTT irrespective of the outcome of their GCT. As previously described [14], this recruitment strategy was specifically designed to generate a cohort of women comprising the full spectrum of glucose tolerance in pregnancy. Participants recruited in pregnancy for this initial cohort returned to the clinical investigation unit at 3-months and 1-year postpartum to undergo repeat cardiometabolic assessment, including evaluation of lipids and glucose tolerance by 2-hour, 75 g OGTT. At 1-year postpartum, they were recruited into the cohort analyzed in the current study, which involved repeat cardiometabolic assessment at 3-years and 5-years postpartum. Of 425 women recruited at 1-year, there were 330 women with singleton pregnancies who completed cardiometabolic characterization at 3- and 5-years postpartum and did not have an intervening pregnancy, thereby comprising the study population evaluated herein. 77 of these women had a normal GCT. The study protocol has been approved by the Mount Sinai Hospital Research Ethics Board and all women provided written informed consent for participation.

### Serial cardiometabolic characterization

On the morning of the antepartum OGTT in late 2nd trimester/early 3rd trimester, participants completed interviewer-administered questionnaires on medical, reproductive and family history. Pre-pregnancy BMI was calculated from their self-reported pre-gravid weight and the measurement of height at the visit. After pregnancy, they returned to the clinical investigation unit for study visits at 3-months, 1-year, 3-years and 5-years postpartum. Each visit involved interviewer-administered questionnaires, bloodwork, and physical examination, including measurement of weight, waist circumference and blood pressure (measured twice 5 min apart by automatic sphygmomanometer (Dinamap Pro 100–400)).

At the study visits at 1-year, 3-years, and 5-years postpartum, physical activity in the preceding year was assessed with the Baecke questionnaire, an established instrument that has been extensively validated in several populations, including women of childbearing age [15, 16]. As previously described [13], this instrument measures total physical activity and its three component domains: occupation-associated activity (work index), sport-related physical activity (sport index), and non-sport leisure-time activity (leisure-time index).

At each of these visits, participants presented in the morning after overnight fast and underwent a 2-hour, 75 g OGTT. During each OGTT, venous blood samples were drawn for measurement of glucose and specific insulin at fasting and at 30-, 60-, and 120-minutes post-challenge, as previously described [14]. These measurements enabled assessment of glucose tolerance status and insulin sensitivity/resistance. Glucose tolerance status (normal glucose tolerance, pre-diabetes, diabetes) was defined according to current Diabetes Canada clinical practice guidelines [17]. Pre-diabetes refers to impaired fasting glucose tolerance (IFG), impaired glucose tolerance (IGT) or combined IFG and IGT [17]. Whole-body insulin sensitivity was assessed with the Matsuda index [18] and insulin resistance (primarily hepatic) was measured with the Homeostasis Model Assessment of insulin resistance (HOMA-IR) [19].

At each of these visits, total cholesterol (Roche CHOL2 reagent), HDL cholesterol (Roche direct HDL reagent, HDLC3) and triglycerides (Roche TRIGL reagent) were measured from fasting serum with the Roche Cobas 6000 c 501 analyzer (Roche Diagnostics, Laval, QC). Lipid measurements were standardized by the Centers for Disease Control and Prevention Lipid Standardization Program (Atlanta, GA). LDL cholesterol was calculated by Friedewald formula. Apolipoprotein-B (apoB), apolipoprotein-A1 (apoA1), and high-sensitivity C-reactive protein (CRP) (N high-sensitivity CRP reagent) were measured with the Siemens Healthcare Diagnostics BN ProSpec (Siemens Healthcare Diagnostics, Mississauga, ON). Total adiponectin was measured by enzyme-linked immunosorbent assay (EMD Millipore, St. Charles, MO).

### Statistical analyses

**Fig. 1:**
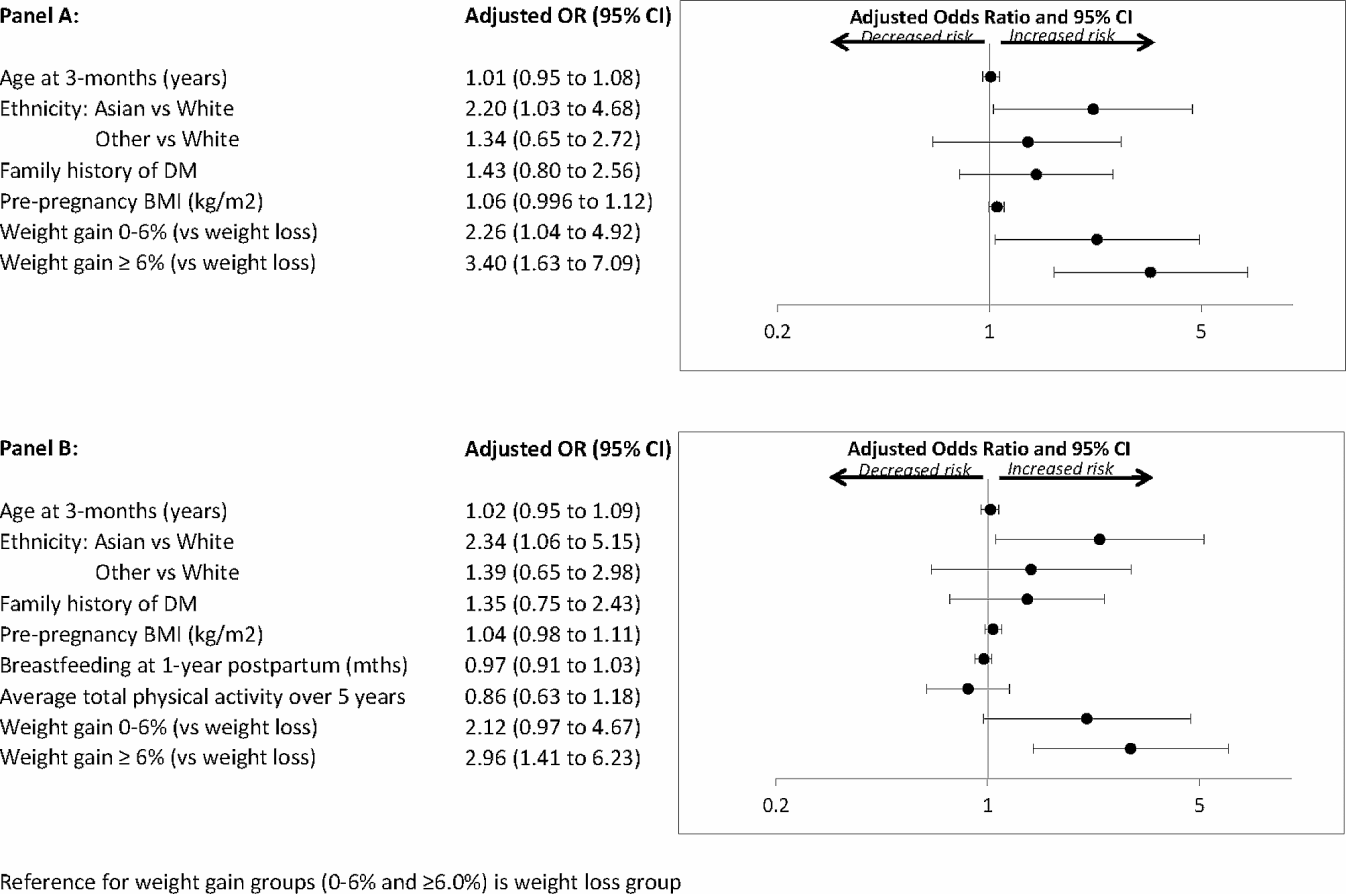
Logistic regression analyses of pre-diabetes/diabetes at 5-years postpartum: **(A)** Model adjusted for age, ethnicity, family history of DM, pre-pregnancy BMI, and % weight gain from pre-pregnancy to 5-yrs postpartum. **(B)** Model further adjusted for duration of breastfeeding in 1st year and average total physical activity at 1-year, 3-years and 5-years

All analyses were performed with SAS 9.4 (SAS Institute, Cary, NC). The study population was stratified into three groups based on the magnitude of weight change between pre-pregnancy and 5-years postpartum as follows: weight gain < 0% (i.e. weight loss), weight gain 0–6%, and weight gain ≥ 6%. Characteristics of the three groups were compared at baseline and at 1-year postpartum (Table 1) and at 3-years and 5-years postpartum (Table 2), respectively. Continuous variables were compared by analysis of variance for those that were normally-distributed and Wilcoxon Rank-Sum non-parametric test for those that were skewed. Categorical variables were compared by Chi-square test or Fisher’s exact test. Multiple linear regression analyses were performed to compare mean adjusted levels of cardiovascular risk factors across the 3 weight change groups at 1-year, 3-years and 5-years postpartum, respectively, after adjustment for age, ethnicity, family history of diabetes, parity, pre-pregnancy BMI, duration of breastfeeding, and time since delivery (Table 3). At each visit, pairwise comparisons were performed across the three weight gain groups, with Bonferroni correction of *P*-values. Linear mixed effect models were constructed to examine whether weight retention group was associated with the respective averages of the cardiovascular risk factors, and whether such associations depended on time. Adjusted covariates were the same as those in the multiple linear regression models. In sensitivity analysis of the linear mixed effect models, weight retention group was replaced by repeated measure of BMI. Multiple logistic regression analyses were performed to determine if weight retention groups were independently associated with pre-diabetes/diabetes at 5-years postpartum, after adjustment for standard clinical risk factors for diabetes (age, ethnicity, family history of DM, pre-pregnancy BMI) (Fig. 1A), and then after further adjustment for duration of breastfeeding and average total physical activity over the 5-years since delivery (Fig. 1B).

**Table 1:** 3-month and 1-year postpartum characteristics of study population, stratified into 3 groups based on magnitude of change in weight between pre-pregnancy and 5-years postpartum, as follows: (i) weight gain < 0% (weight loss); (ii) weight gain 0–6%; and (iii) weight gain ≥ 6%

			Weight Loss		Weight Gain 0–6%		Weight Gain ≥ 6%		
**At 3-months Postpartum**			**(** *n* ** = 100)**		**(** *n* ** = 110)**		**(** *n* ** = 120)**		**p**
Time since delivery (months)			3.2 (3.0-3.6)		3.1 (3.0-3.4)		3.1 (2.9–3.6)		0.72
Age (years)			35.6 ± 4.2		36.1 ± 4.4		35.5 ± 4.2		0.54
Ethnicity:									0.03
White n(%)			75 (75%)		83(75.5%)		75 (62.5%)		
Asian n(%)			14 (14%)		14(12.7%)		15 (12.5%)		
Other n(%)			11 (11%)		13 (11.8%)		30 (25%)		
Family history of DM n(%)			60 (60%)		71 (64.6%)		77 (64.2%)		0.75
Parity									0.89
1 n(%)			48 (48%)		60 (54.6%)		60 (50%)		
2 n(%)			43 (43%)		40 (36.4%)		48(40%)		
>2 n(%)			9 (9%)		10 (9.0%)		12 (10%)		
Pre-pregnancy BMI (kg/m^2^)			26.9 ± 5.6		24.2 ± 4.1		24.7 ± 4.3		**< 0.0001**
GDM n (%)			39 (39%)		37 (33.6%)		35(29.2)		0.31
Breastfeeding (months)			3.0 (3.0–4.0)		3.0 (2.0–3.0)		3.0 (2.5-3.0)		0.23
BMI (kg/m^2^)			27.1 ± 5.0		25.5 ± 4.0		26.6 ± 4.3		**0.04**
Waist circumference (cm)			92.0 ± 12.7		88.8 ± 10.9		88.9 ± 9.8		0.076
**At 1-year Postpartum**									
Time since delivery (months)			12 (12–13)		12 (12–13)		12 (12–13)		0.34
Breastfeeding (months)			9 (5.5–12)		10.5 [6–12]		9 [4–12]		0.78
Total physical activity			8.0 ± 1.4		8.3 ± 1.4		8.1 ± 1.3		0.51
Sport index			2.2 ± 0.7		2.3 ± 0.8		2.1 ± 0.7		0.18
Leisure time index			3.0 ± 0.5		3.1 ± 0.6		3.1 ± 0.5		0.30
Work index			2.8 ± 0.6		2.8 ± 0.6		2.9 ± 0.6		0.70
BMI (kg/m^2^)			26.3 ± 5.7		24.4 ± 4.1		26.3 ± 4.8		**0.005**
Waist circumference (cm)			89.6 ± 14.6		84.5 ± 9.9		87.9 ± 12.1		**0.01**
Systolic BP (mm Hg)			109.0 ± 11.8		109.2 ± 12.7		108.9 ± 11.3		0.98
Diastolic BP (mm Hg)			65.3 ± 7.6		65.0 ± 9.8		64.4 ± 8.7		0.77
Total cholesterol (mmol/l)			4.6 ± 0.8		4.7 ± 0.8		4.7 ± 0.8		0.43
LDL cholesterol (mmol/l)			2.7 ± 0.8		2.7 ± 0.8		2.8 ± 0.7		0.79
HDL cholesterol (mmol/l)			1.4 ± 0.4		1.5 ± 0.4		1.4 ± 0.3		**0.04**
Triglycerides (mmol/)			0.9 (0.7–1.2)		0.8 (0.6–1.3)		0.9 (0.7–1.2)		0.80
apoB (g/L)			0.8 ± 0.2		0.8 ± 0.2		0.8 ± 0.2		0.99
apoB:apoA1			0.6 ± 0.2		0.5 ± 0.2		0.6 ± 0.1		0.33
Fasting glucose (mmol/L)			4.7 ± 0.4		4.8 ± 0.5		4.8 ± 0.5		0.49
2-hour glucose (mmol/L)			6.1 ± 1.6		6.3 ± 1.7		6.3 ± 1.8		0.53
HOMA-IR			1.3 (0.9–2.3)		1.2 (0.7–1.7)		1.3 (0.7-2.0)		0.13
Matsuda index			8.1 (4.9–11.3)		8.9 (5.1–13.6)		8.2 (4.9–13.1)		0.31
CRP (mg/L)			1.1 (0.6–3.2)		1.0 (0.5–2.2)		1.2 (0.5-3.0)		0.54
Adiponectin (ug/ml)			9.2 ± 3.9		9.7 ± 3.7		9.0 ± 3.9		0.33

Continuous data are presented as mean ± standard deviation (if normal distribution) or median followed by interquartile range in parentheses (if skewed distribution). Categorical variables are presented as absolute number followed by percentage in parentheses

Bold indicates *p* < 0.05

**Table 2:** Cardiovascular risk factor profiles of the weight change groups at 3-years and 5-years postpartum

			Weight Loss		Weight Gain 0–6%		Weight Gain ≥ 6%		
**At 3-years Postpartum**			**(** *n* ** = 100)**		**(** *n* ** = 110)**		**(** *n* ** = 120)**		**p**
Time since delivery (months)			33 (26–38)		34 (27–40)		32 (25–41)		0.74
Total physical activity			8.6 ± 1.0		8.6 ± 0.9		8.5 ± 1.0		0.65
Sport index			3.0 ± 0.6		3.0 ± 0.5		2.9 ± 0.5		0.22
Leisure time index			3.0 ± 0.6		3.0 ± 0.5		3.0 ± 0.6		0.90
Work index			2.6 ± 0.5		2.6 ± 0.6		2.7 ± 0.5		0.52
BMI (kg/m^2^)			25.8 ± 5.4		24.7 ± 4.5		26.9 ± 5.0		**0.005**
Waist circumference (cm)			88.1 ± 13.0		86.0 ± 11.7		89.8 ± 1.7		0.06
Systolic BP (mm Hg)			108.3 ± 11.3		111.1 ± 13.4		109.6 ± 12.5		0.26
Diastolic BP (mm Hg)			65.1 ± 9.1		66.7 ± 9.9		66.9 ± 10.1		0.36
Total cholesterol (mmol/l)			4.4 ± 0.9		4.6 ± 0.7		4.6 ± 0.8		0.18
LDL cholesterol (mmol/l)			2.5 ± 0.8		2.6 ± 0.7		2.6 ± 0.7		0.60
HDL cholesterol (mmol/l)			1.4 ± 0.3		1.6 ± 0.4		1.4 ± 0.3		**0.03**
Triglycerides (mmol/)			0.9 (0.7–1.1)		0.9 (0.7–1.2)		1.0 (0.7–1.3)		**0.03**
apoB (g/L)			0.8 ± 0.2		0.8 ± 0.2		0.8 ± 0.2		0.31
apoB:apoA1			0.5 ± 0.2		0.5 ± 0.2		0.5 ± 0.1		0.24
Fasting glucose (mmol/L)			4.6 ± 0.5		4.7 ± 0.5		4.8 ± 0.5		**0.03**
2-hour glucose (mmol/L)			6.4 ± 1.6		6.3 ± 1.9		6.6 ± 2.0		0.39
HOMA-IR			1.3 (0.8–2.1)		1.3 (0.8–1.8)		1.5 (1.0-2.3)		**0.02**
Matsuda index			8.4 (4.8–12.2)		8.4 (5.5–11.2)		6.4 (3.8–10.0)		**0.004**
CRP (mg/L)			1.0 (0.4–2.1)		1.0 (0.4-2.0)		1.2 (0.5–3.4)		0.09
Adiponectin (ug/ml)			9.9 ± 4.4		10.4 ± 4.5		8.9 ± 4.2		**0.04**
**At 5-years Postpartum**									
Time since delivery (months)			55 (49–67)		56 (51–65)		57 (51–71)		0.28
Total physical activity			8.5 ± 0.9		8.5 ± 1.0		8.4 ± 1.1		0.66
Sport index			2.9 ± 0.5		2.9 ± 0.5		2.9 ± 0.6		0.91
Leisure time index			3.0 ± 0.5		3.0 ± 0.6		3.0 ± 0.6		0.83
Work index			2.6 ± 0.5		2.5 ± 0.5		2.5 ± 0.5		0.39
BMI (kg/m^2^)			25.2 ± 5.3		24.8 ± 4.1		27.7 ± 5.0		**< 0.0001**
Waist circumference (cm)			86.3 ± 12.1		86.4 ± 10.4		91.7 ± 13.2		**0.0008**
Systolic BP (mm Hg)			108.6 ± 14.7		110.4 ± 12.7		110.6 ± 12.8		0.49
Diastolic BP (mm Hg)			65.9 ± 10.3		65.9 ± 9.4		67.0 ± 9.5		0.60
Total cholesterol (mmol/l)			4.4 ± 0.9		4.6 ± 0.7		4.6 ± 0.7		**0.04**
LDL cholesterol (mmol/l)			2.4 ± 0.8		2.4 ± 0.7		2.6 ± 0.6		0.12
HDL cholesterol (mmol/l)			1.6 ± 0.4		1.7 ± 0.4		1.5 ± 0.4		**0.009**
Triglycerides (mmol/)			0.9 (0.7–1.2)		1.0 ( 0.8–1.4)		1.1 (0.8–1.5)		**0.003**
apoB (g/L)			0.77 ± 0.21		0.80 ± 0.20		0.84 ± 0.17		**0.03**
apoB:apoA1			0.5 ± 0.2		0.5 ± 0.2		0.6 ± 0.1		0.07
Fasting glucose (mmol/L)			4.6 ± 0.5		4.7 ± 0.5		4.8 ± 0.6		**0.03**
2-hour glucose (mmol/L)			6.4 ± 1.9		6.7 ± 2.1		7.1 ± 2.3		**0.04**
HOMA-IR			1.2 (0.8-2.0)		1.2 (0.9–1.9)		1.6 (1.2–2.4)		**0.0002**
Matsuda index			8.3 (4.9–12.2)		7.6 (5.1–10.5)		5.9 (3.6–9.4)		**0.0004**
CRP (mg/L)			1.0 (0.4–2.6)		1.0 (0.4–2.1)		1.4 (0.5–3.8)		0.12
Adiponectin (ug/ml)			11.9 ± 5.4		12.0 ± 5.4		10.1 ± 5.4		**0.01**

Continuous data are presented as mean ± standard deviation (if normal distribution) or median followed by interquartile range in parentheses (if skewed distribution)

Bold indicates *p* < 0.05

**Table 3:** Mean adjusted levels of cardiovascular risk factors in the 3 weight change groups at 1-year, 3-years, and 5-years postpartum, respectively, after adjustment for age, ethnicity, family history of DM, parity, pre-pregnancy BMI, duration of breastfeeding, and time since delivery ^*a*^ *denotes P < 0.05 for weight gain ≥ 6.0% vs. weight loss;* ^*b*^ *denotes P < 0.05 for weight gain 0–6% vs. weight loss;* ^*c*^ *denotes P < 0.05 for weight gain ≥ 6.0% vs. weight gain 0–6%*

			Weight Loss		Weight Gain 0–6%		Weight Gain ≥6%		
**Cardiovascular Risk Factor**			**(** *n* ** = 100)**		**(** *n* ** = 110)**		**(** *n* ** = 120)**		**P**
Triglycerides (mmol/l):									
At 1-year			0.90 ± 0.05		1.00 ± 0.05		0.98 ± 0.05		0.2
At 3-years			0.84 ± 0.04		0.92 ± 0.04		0.98 ± 0.04 ^a^		**0.02**
At 5-years			0.90 ± 0.05		1.05 ± 0.05 ^b^		1.11 ± 0.05 ^a^		**0.001**
HDL cholesterol (mmol/l):									
At 1-year			1.45 ± 0.04		1.48 ± 0.04		1.42 ± 0.04		0.44
At 3-years			1.44 ± 0.04		1.46 ± 0.04		1.41 ± 0.04		0.51
At 5-years			1.61 ± 0.05		1.62 ± 0.05		1.49 ± 0.04 ^c^		**0.02**
LDL cholesterol (mmol/l):									
At 1-year			2.56 ± 0.10		2.67 ± 0.10		2.69 ± 0.09		0.47
At 3-years			2.42 ± 0.10		2.55 ± 0.10		2.56 ± 0.08		0.36
At 5-years			2.26 ± 0.10		2.48 ± 0.09		2.59 ± 0.08 ^a^		**0.01**
apoB (g/L):									
At 1-year			0.78 ± 0.03		0.82 ± 0.02		0.81 ± 0.02		0.47
At 3-years			0.72 ± 0.02		0.77 ± 0.02		0.77 ± 0.02		0.13
At 5-years			0.74 ± 0.03		0.82 ± 0.03^b^		0.84 ± 0.02^a^		**0.003**
Matsuda index:									
At 1-year			6.86 ± 0.48		6.85 ± 0.47		6.92 ± 0.43		0.99
At 3-years			6.96 ± 0.44		6.02 ± 0.38		5.26 ± 0.29 ^a^		**0.0006**
At 5-years			7.28 ± 0.49		6.02 ± 0.38 ^b^		5.06 ± 0.29 ^a,c^		**< 0.0001**
HOMA-IR:									
At 1-year			1.37 ± 0.09		1.35 ± 0.09		1.38 ± 0.08		0.95
At 3-years			1.34 ± 0.09		1.59 ± 0.10		1.75 ± 0.10 ^a^		**0.002**
At 5-years			1.21 ± 0.08		1.44 ± 0.09		1.81 ± 0.10 ^a,c^		**< 0.0001**
Fasting glucose (mmol/l):									
At 1-year			4.70 ± 0.06		4.78 ± 0.06		4.83 ± 0.05		0.18
At 3-years			4.64 ± 0.06		4.73 ± 0.06		4.84 ± 0.05 ^a^		**0.01**
At 5-years			4.63 ± 0.07		4.74 ± 0.07		4.82 ± 0.06		0.07
CRP (mg/L):									
At 1-year			1.01 ± 0.14		1.10 ± 0.15		1.21 ± 0.15		0.55
At 3-years			0.82 ± 0.11		1.01 ± 0.14		1.27 ± 0.15 ^a^		**0.02**
At 5-years			0.77 ± 0.12		1.04 ± 0.16		1.34 ± 0.18 ^a^		**0.01**

Bold indicates *p* < 0.05

## Results

The 425 women (ethnicity: white 67.2%, Asian 14.6%, other 18.2%) recruited at 1-year postpartum had mean age 35.5 ± 4.3 years, pre-pregnancy BMI 25.1 ± 5.0 kg/m^2^, with median 12.1 months since delivery (interquartile range 11.7–12.9) and median 10 months breastfeeding (interquartile range 5–12). 50.4% were primiparous and 63.7% had a family history of diabetes. Compared to those who did not complete the visits at 3- and 5-years postpartum, the 330 women who completed these visits were slightly older (mean age 35.7 ± 4.3 vs. 34.8 ± 4.3 years, *p = 0.047), with no significant differences in ethnicity, family history of diabetes, parity, pre-pregnancy BMI, duration of breastfeeding, or time since delivery (data not shown).*

The 330 study participants were stratified into the following three groups based on their weight change between pre-pregnancy and 5-years postpartum: (i) women with weight gain < 0% (i.e. weight loss; *n* = 100), (ii) those with weight gain 0–6% (*n* = 110), and (iii) women with weight gain ≥ 6% (*n* = 120). As shown in Table 1, there were no significant differences between the groups in age, ethnicity, family history of diabetes, parity, and prevalence of GDM in the recent pregnancy. Of note, pre-pregnancy BMI was higher in the weight loss group (mean 26.9 ± 5.6 kg/m^2^) than in the weight gain 0–6% (mean 24.2 ± 4.1 kg/m^2^) and weight gain ≥ 6% group (mean 24.7 ± 4.3 kg/m^2^) (overall *p* < 0.0001), and this comparative difference persisted at 3-months postpartum (*p* = 0.04).

At 1-year postpartum (Table 1), BMI continued to differ between the groups (overall *p* = 0.005), but now it was highest in both the weight loss and weight gain ≥ 6% groups (both mean BMI 26.3 kg/m^2^). Waist circumference was highest in the weight loss group (*p* = 0.01), with no differences between the groups in duration of breastfeeding and physical activity in the preceding year. Of note, there were no significant differences in cardiovascular risk factors at 1-year postpartum, including blood pressure, lipid profile, fasting glucose, 2-hour glucose, HOMA-IR, Matsuda index, CRP and adiponectin.

By 3-years postpartum (Table 2), the weight gain ≥ 6% group had the highest BMI (mean 26.9 kg/m^2^) (overall *p* = 0.005), coupled with the largest waist circumference (mean 89.8 cm) (overall *p* = 0.06). Again, there were no differences in physical activity in the preceding year. However, the weight gain ≥ 6% group now had lower HDL cholesterol (*p* = 0.03), the highest triglycerides (*p* = 0.03), highest fasting glucose (*p* = 0.03), poorest insulin sensitivity (Matsuda index: *p* = 0.004; HOMA-IR: *p* = 0.02), and lowest adiponectin (*p* = 0.04). These emergent differences in cardiovascular risk factors were further amplified at 5-years postpartum, at which time both BMI (mean 27.7 kg/m^2^) and waist circumference (mean 91.7 cm) were highest in the weight gain ≥ 6% group (overall *p* < 0.0001 and *p* = 0.0008, respectively). Indeed, compared to their peers, the women comprising this group now exhibited the highest total cholesterol (*p* = 0.04), apoB (*p* = 0.03), triglycerides (*p* = 0.003), fasting glucose (*p* = 0.03), 2-hour glucose on the OGTT (*p* = 0.04), and HOMA-IR (*p* = 0.0002), coupled with the lowest Matsuda index (*p* = 0.0004), HDL cholesterol (*p* = 0.009) and adiponectin (*p* = 0.01).

We next compared mean adjusted levels of cardiovascular risk factors in the three groups at each of 1-year, 3-years, and 5-years postpartum, respectively, after adjustment for age, ethnicity, family history of diabetes, parity, pre-pregnancy BMI, duration of breastfeeding and time since delivery (Table 3). These analyses revealed that, after covariate adjustment, there was stepwise worsening (from the weight loss group to weight gain 0–6% to weight gain ≥ 6%) of the following CV risk factors at 5-years: triglycerides (*p* = 0.001), HDL (*p* = 0.02), LDL (*p* = 0.01), apoB (*p* = 0.003), Matsuda index (*p* < 0.0001), HOMA-IR (*p* < 0.0001), fasting glucose (*p* = 0.07), and C-reactive protein (*p* = 0.01). Moreover, none of these differences were present at 1-year postpartum. Instead, these significant differences in mean adjusted cardiovascular risk factors progressively emerged over time at 3- and 5-years (Table 3). These findings were unchanged on sensitivity analyses adjusting for the area-under-the-glucose-curve on the OGTT in pregnancy (data not shown). Linear mixed models also revealed that significant differences in the averages of cardiovascular risk factors across the weight retention groups depended on the time since delivery, as follows: triglycerides (interaction effect between weight group and time, *p = 0.04), HDL (p = 0.01), apoB (p = 0.002), Matsuda index (p < 0.0001) and HOMA-IR (p = 0.0003). On sensitivity analyses in which weight retention group was replaced with repeated measure BMI, the change in BMI showed significant effect on the average rate of change in triglycerides (interaction effect between BMI and time, p = 0.04) and HDL (p = 0.01).*

Having identified that greater postpartum weight retention tracks with the early evolution of an adverse cardiovascular risk profile over the first 5-years after pregnancy, we next considered its implications for dysglycemia (i.e. pre-diabetes/diabetes). At 5-years postpartum, 77 women had dysglycemia, of which the vast majority was pre-diabetes (*n* = 65). On logistic regression analyses adjusted for age, ethnicity, family history of diabetes, and pre-pregnancy BMI, postpartum weight gain ≥ 6% emerged as an independent predictor of pre-diabetes/diabetes at 5-years (adjusted OR = 3.40, 95%CI: 1.63–7.09) (Fig. 1A). These findings were unchanged with further adjustment for duration of breastfeeding and average total physical activity over the preceding 5-years (adjusted OR = 2.96, 95%CI: 1.41–6.23) (Fig. 1B). Similarly, the findings were unchanged with further adjustment for parity (data not shown).

## Discussion

In this report, we demonstrate the emergence over time of an adverse cardiovascular risk factor profile in the first 5-years after pregnancy in women with postpartum weight retention. Specifically, compared to women with no such retention, those with weight gain 0–6% and ≥ 6%, respectively, initially showed no differences in traditional (lipids, glycemia, blood pressure) and non-traditional cardiovascular risk factors (apoB, CRP, adiponectin) at 1-year postpartum. However, at 3-years and 5-years postpartum, adjusted analyses revealed stepwise worsening of triglycerides, HDL, LDL, apoB, Matsuda index, HOMA-IR, fasting glucose, and CRP from the weight loss group to weight gain 0–6% to the weight gain ≥ 6% group. Moreover, on logistic regression analyses, weight gain ≥ 6% was an independent predictor of pre-diabetes/diabetes at 5-years. It thus emerges that postpartum weight retention predicts glucose intolerance and trajectories of worsening cardiovascular risk factors over the first 5-years after delivery.

Pregnancy is unique in representing a normal life event during which body weight may be expected to increase by 20% or more over 9-months. After delivery, there are many practical and logistical factors that may serve as barriers that compromise maternal capacity to return to pre-pregnancy weight. Indeed, factors such as lack of sleep, limited social support, emotional stress and the demands of childcare can make it difficult for a new mother to focus on her own care and the healthy lifestyle habits that may facilitate restoration of her pre-gravid weight [20–22]. Previous studies have suggested that a typical pattern is that most women will retain a degree of gestational weight in the early postpartum months, followed by some weight loss thereafter [13, 23, 24]. We have previously noted that the small proportion of women who do not lose weight between 3- and 12-months after delivery have a more adverse vascular risk factor profile at 1-year postpartum than their peers who achieve weight reduction during this time [13]. While the latter comprise the majority of women, a common scenario is that the weight loss ultimately achieved in the years after delivery does not fully offset that which is gained during gestation. Accordingly, it has been postulated that the cumulative effect of such weight retention from each of the pregnancies during her lifetime may contribute to a woman’s future risk of cardiovascular disease [10, 12, 13].

In the current study, we sought to provide evidence supportive of this hypothesis by evaluating the impact of postpartum weight retention on the trajectories of cardiovascular risk factors over the first 5-years after pregnancy. By systematically measuring traditional and non-traditional risk factors at 1-, 3- and 5-years, we demonstrate the evolution of differential cardiovascular risk profiles over time in women who retain weight after pregnancy as compared to those who do not. Two points in particular should be noted from these data. First, the women with postpartum weight gain ≥ 6% began accruing an adverse vascular risk factor profile within 5-years after delivery despite entering pregnancy with a relatively modest mean pre-gravid BMI of 24.7 kg/m^2^. Second, the impact of exposure over time to this change in body weight is apparent. Specifically, while cardiovascular risk factors were initially indistinct between the 3 groups at 1-year postpartum, this relative similarity gave way to emergent differences at 3-years that were then more pronounced at 5-years, indicative of differential trajectories over time. This evolution is conceptually reminiscent of current thinking around the potentially transient nature of metabolically healthy obesity, wherein longer follow-up reveals transition to metabolically unhealthy status that may ultimately explain the increased long-term cardiovascular mortality observed in this patient population [25]. Indeed, the stepwise accrual of cardiovascular risk factors between 1-, 3- and 5-years after pregnancy in women with postpartum weight retention may be reflecting a similar transition with exposure over time. Moreover, when coupled with the recognition that $\sim$ 80% of women will have a pregnancy at some point in their lifetime [26], these data highlight the potential public health importance of addressing postpartum weight retention as a strategy for mitigating the long-term incidence of cardiometabolic disease in women.

The American College of Obstetrics and Gynecology (ACOG) has recently emphasized postpartum care as a clinical opportunity for improving the long-term health of women [27]. In this context, the current findings highlight the potential value of targeting postpartum weight retention. Indeed, limited clinical trials of postpartum lifestyle intervention have reported reduction in weight retention [28, 29], although the aforementioned logistical factors that may limit the capacity of new mothers to focus on self-care can similarly compromise weight management interventions. Conversely, another window of opportunity for intervention may be prior to conception, consistent with a recent position statement from the International Federation of Gynecology and Obstetrics (FIGO) recommending optimization of preconception care [30]. Optimizing maternal lifestyle and weight status before pregnancy may reduce gestational weight gain and thereby limit postpartum weight retention. Of note, this hypothesis is being tested by the Healthy Life Trajectories Initiative (HeLTI) consortium, which is currently conducting 4 harmonized clinical trials of culturally-tailored, preconception lifestyle interventions in women planning pregnancies in South Africa, India, China and Canada [31–34].

A limitation of this study is that the dietary patterns of participants were not assessed. Thus, though physical activity did not differ between the groups, we cannot determine the potential impact that dietary habits may have had on the observed postpartum weight retention. Secondly, the observational design precludes definitive ascertainment of causality in the observed associations. In addition, pre-pregnancy weight was determined by participant self-report during the antepartum OGTT (albeit prior to ascertainment of glucose tolerance status). Nevertheless, while recruitment in pregnancy may have yielded some misclassification of women in the weight gain groups, the consistency of the stepwise progression of cardiovascular risk factors across the 3 groups over time is supportive of the reliability of the findings reported herein.

In conclusion, postpartum weight retention is associated with the progressive emergence of an adverse cardiovascular profile over the first 5-years after pregnancy. Compared to their peers with no such retention, women with weight gain 0–6% and ≥ 6%, respectively, initially exhibit no differences in traditional and non-traditional cardiovascular risk factors at 1-year postpartum. By 3-years and 5-years postpartum, however, there is a progressive stepwise worsening of triglycerides, HDL, LDL, apoB, Matsuda index, HOMA-IR, fasting glucose, and CRP from women with no gestational weight retention to those with weight gain 0–6% to those with weight gain ≥ 6%. Moreover, on logistic regression analyses, weight gain ≥ 6% emerges as an independent predictor of pre-diabetes/diabetes at 5-years. Postpartum weight retention thus predicts the early evolution of cardiovascular risk over the first 5-years after pregnancy, consistent with its postulated contribution to the likelihood of future vascular disease in women.

## Data Availability

De-identified data can be made available under restricted access from the corresponding author, for academic purposes, subject to a material transfer agreement and approval of the Mount Sinai Hospital Research Ethics Board.

## Abbreviations

apoA1: Apolipoprotein–A1
apoB: Apolipoprotein–B
CRP: C–reactive protein
CVD: Cardiovascular disease
GCT: Glucose challenge test
GDM: Gestational diabetes
HDL: High–density–lipoprotein
HOMA: IR–Homeostasis Model Assessment of Insulin Resistance
LDL: Low–density–lipoprotein
OGTT: Oral glucose tolerance test
T2DM: Type 2 diabetes
